# Phenothiourea Sensitizes Zebrafish Cranial Neural Crest and Extraocular Muscle Development to Changes in Retinoic Acid and IGF Signaling

**DOI:** 10.1371/journal.pone.0022991

**Published:** 2011-08-19

**Authors:** Brenda L. Bohnsack, Donika Gallina, Alon Kahana

**Affiliations:** Department of Ophthalmology and Visual Sciences, Kellogg Eye Center, University of Michigan, Ann Arbor, Michigan, United States of America; Laboratoire Arago, France

## Abstract

1-phenyl 2-thiourea (PTU) is a tyrosinase inhibitor commonly used to block pigmentation and aid visualization of zebrafish development. At the standard concentration of 0.003% (200 µM), PTU inhibits melanogenesis and reportedly has minimal other effects on zebrafish embryogenesis. We found that 0.003% PTU altered retinoic acid and insulin-like growth factor (IGF) regulation of neural crest and mesodermal components of craniofacial development. Reduction of retinoic acid synthesis by the pan-aldehyde dehydrogenase inhibitor diethylbenzaldehyde, only when combined with 0.003% PTU, resulted in extraocular muscle disorganization. PTU also decreased retinoic acid-induced teratogenic effects on pharyngeal arch and jaw cartilage despite morphologically normal appearing PTU-treated controls. Furthermore, 0.003% PTU in combination with inhibition of IGF signaling through either morpholino knockdown or pharmacologic inhibition of tyrosine kinase receptor phosphorylation, disrupted jaw development and extraocular muscle organization. PTU in and of itself inhibited neural crest development at higher concentrations (0.03%) and had the greatest inhibitory effect when added prior to 22 hours post fertilization (hpf). Addition of 0.003% PTU between 4 and 20 hpf decreased thyroxine (T4) in thyroid follicles in the nasopharynx of 96 hpf embryos. Treatment with exogenous triiodothyronine (T3) and T4 improved, but did not completely rescue, PTU-induced neural crest defects. Thus, PTU should be used with caution when studying zebrafish embryogenesis as it alters the threshold of different signaling pathways important during craniofacial development. The effects of PTU on neural crest development are partially caused by thyroid hormone signaling.

## Introduction

Zebrafish (*Danio rerio*) are small fresh-water tropical fish that have gained popularity as a vertebrate model for biological research over the past 20 years [1]. Zebrafish are particularly amenable for studying organismal development because of their small size, extracorporeal development, and accessibility for forward and reverse genetics. Unlike mammalian models, *in vivo* imaging of live zebrafish embryos, combined with transgenic approaches, can yield real-time viewing of development [2]. Through the use of real time experiments as well as standard molecular techniques (ie. *in situ* hybridization and immunostaining) employed at specific time points, zebrafish models have yielded significant insights into embryogenesis, organogenesis, tissue regeneration and pathogenesis.

For optimal analysis, zebrafish embryos should remain transparent to achieve optical clarity during *in vivo* imaging and easy detection of whole mount color reagents. Pigmentation begins around 24 hours post fertilization (hpf; 5 prim stage) in the retinal pigment epithelium and then extends throughout the skin by 48 hpf [3], [4]. Most commonly, embryos are treated with the tyrosinase inhibitor 1-phenyl 2-thiourea (PTU) to block endogenous melanogenesis [3], [5]. The tyrosinase enzyme is required to form the two intermediates (L-dopa and dopaquinone) in the conversion from tyrosine to melanin [6]. Addition of PTU prior to 24 hpf effectively but reversibly inhibits pigmentation until approximately 120 hpf.

For the purpose of inhibiting pigmentation, PTU is typically used at a concentration of 0.003% (200 µM) [7] which reportedly has no effect on zebrafish embryonic heart rate [1], catecholamine synthesis, or xanthophore and iridophore differentiation [8]. However, at this concentration, PTU added at the 28 somite-stage has been demonstrated to cause mild teratogenesis (posterior malformation and protruding lower snout), delayed hatching, and mortality by 120 hpf [3] in approximately one-third of embryos. In fact, Karlsson and colleagues found that PTU at a concentration of 70 µM that is added to the media at the 28 somitic stage minimizes mortality and teratogenic effects, but still maintains transparency. Furthermore, PTU at a concentration of 0.003% has been reported to inhibit thyroid function similar to methimazole and potassium percholorate, which are well-known goitrogens [5], [9]. It is unknown whether the toxic effects of PTU are through inhibition of thyroid hormone. Despite these findings, the standard concentration of PTU cited in most protocols remains 0.003% (200 µM).

To bypass the need for PTU, additional strategies such as the use of zebrafish strains that have decreased levels or absence of pigment have been employed. One example is the *roy orbison* (*roy*) strain which is a naturally occurring mutant that has decreased melanocytes and lacks autofluorescence due to absence of iridophores [10]. By comparing phenotypes in a wildtype background in the presence of 0.003% PTU to phenotypes in a *roy* strain, we found that PTU interacts with the teratogen retinoic acid as well as other signaling pathways such as insulin-like growth factor (IGF) to influence craniofacial and extraocular muscle development. Importantly, while 0.003% PTU treatment alone (control treatment) resulted in embryos that lacked pigment but were otherwise morphologically normal, a higher concentration of PTU, or earlier treatment, inhibited cranial neural crest development.

From these studies we conclude that the use of PTU in combination with pharmacologic treatments or genetic manipulations of zebrafish embryos, even at 0.003%, can mask or alter phenotypes, and particularly those affecting neural crest and craniofacial development.

## Materials and Methods

### Zebrafish care, mutants and transgenics

Zebrafish (*Danio rerio*) were raised in a laboratory breeding colony on a 14 hour light/10 hour dark cycle. Embryos were maintained at 28.5 degrees Celsius and staged as described [4]. Tg (*sox10::EGFP*) strain was the generous gift of Thomas Schilling [11], [12]. The Tg (*fli::EGFP*) strain was obtained from ZIRC (Eugene, OR). The Tg(a-*actin*::EGFP) strain was a generous gift of Dr. Simon Hughes, [13]. These transgenic strains were crossed into the *roy* background [10], which was the generous gift of Rachel Wong, to decrease endogenous fluorescence. The protocols have met guidelines established by the University of Michigan Committee on the Use and Care of Animals.

### Phenylthiourea treatment

1-phenyl 2-thiourea (PTU; Sigma, St. Louis, MO) was dissolved in water to 0.3%, and diluted in embryo media to the indicated concentrations (0.003% to 0.03%) at specific developmental time points (6 to 22 hpf).

### Morpholinos Oligonucleotide (MO) Injections

Antisense oligonucleotide morpholinos (MOs) were synthesized by Gene Tools, LLC (Cowallis, OR) and reconstituted in de-ionized water. MO sequences for *igf1ra*, *igf1rb*, and standard control (*globin*) were previous published [14]. The concentration of MO for each gene that yielded consistent and reproducible phenotype was determined. MO sequences were lissamine-tagged for fluorescent tracking. 1 nl of MO (0.1–0.25 mM) was injected into the yolk of 1–2 cell stage embryos.

Embryos were analyzed with a Leica M205FA combi-scope (Leica, Germany) using a Leica DFC290 (brightfield imaging) and Hamamatsu ORCA-ER (fluorescent imaging) cameras (Hamamatsu, Japan).

### Pharmacological Treatment of Embryos

All-trans retinoic acid (Sigma), diethylbenzaldehyde (DEAB; Sigma), picropodophyllin (PPP; Calbiochem, La Jolla, CA), triiodothyronine (T3; Sigma), or thyroxine (T4; Sigma), were dissolved in dimethylsulfoxide (DMSO) to 1000× of final concentration. The pharmacologic agents were added to embryo media to their final concentrations (retinoic acid 0.1 nM to 1 µM; DEAB 10–20 µM; PPP 2–10 µM; T3 100 nM; T4 100 nM) at the indicated time. 0.1% DMSO served as control. Exogenous treatment was initiated at the developmental time as indicated. At the cessation of treatment, the embryos were washed in embryo media 3 times and then raised in fresh embryo media until the designated time. Embryo media was changed every 24 hours with fresh pharmacologic agent as indicated until the embryos were harvested.

### Wholemount Immunostaining for Thyroxine

Embryos were harvested at 96 hpf and fixed in 2% trichloroacetic acid for 3 hours at room temperature. Embryos were then washed in PBS/1% triton and blocked in 10% normal goat serum in PBS/1% triton for 2 hours. Embryos were incubated with rabbit anti-thyroxine (T4) polyclonal antibody (1∶1000; MP Biochemicals, Solon, OH) in 1% normal goat serum in PBS/1% triton overnight at 4 degrees Celsius. Embryos were washed multiple times with PBS/1% triton and then incubated overnight at 4 degrees Celsius with goat anti-rabbit IgG conjugated with Cy3 (Abcam, Cambridge, MA) in 1% normal goat serum in PBS/1% triton. Embryos were washed multiple times with PBS/1% triton and then analyzed microscopically.

## Results

### PTU Modulates Retinoic Acid Regulation of Craniofacial Development

**Figure 1:**
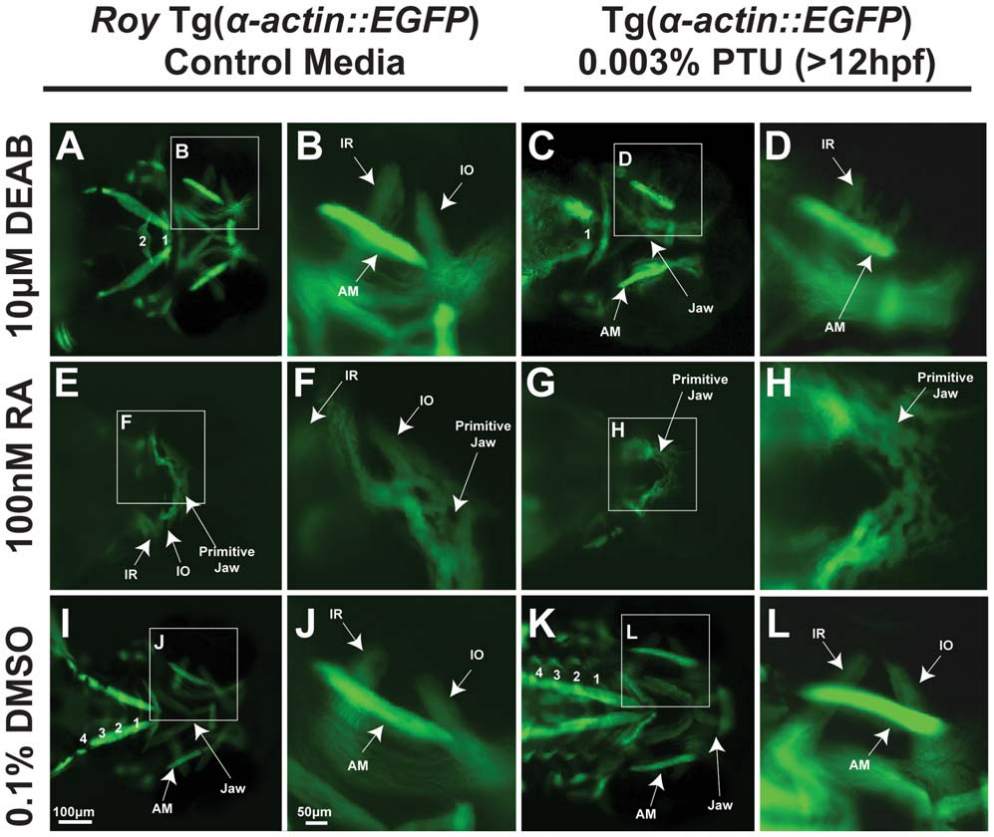
PTU modulates retinoic acid regulation of craniofacial muscles. 72 hpf Tg(*α-actin::EGFP*) embryos (ventral view) were treated with 10 µM DEAB (A–D) at 24 hpf, 100 nM retinoic acid (RA) (E–F) at 28 hpf, , or 0.1% dimethylsulfoxide (DMSO; I–L) in the *roy* background (in the absence of PTU; A, B, E, F, I, J) or in the presence of 0.003% PTU which was added to the media at 12 hpf (C, D, G, H, K, L). 10 µM DEAB in the presence of PTU caused thickening of the inferior rectus (C, D) and absence of the inferior oblique (IO) compared to treatment with DEAB in the *roy* background (absence of PTU; A, B). In addition, only 1 pharyngeal arch (PA) was present in embryos treated with DEAB and PTU (C). Embryos treated with DEAB alone had 2 pharyngeal arches (A) while the control embryos had 4 pharyngeal arches (I). Exogenous retinoic acid inhibited craniofacial muscle development which was worse in the absence of PTU (G. H compared to E, F). AM, anterior mandibulae.

Retinoic acid is well-recognized for its influence on craniofacial development both as an essential morphogen as well as a teratogen [15], [16], [17], [18], [19]. Using transgenic zebrafish strains that express GFP in neural crest or differentiated muscle (Tg(*sox10::EGFP*) and Tg(*α-actin::EGFP*), respectively) [20] we found that 0.003% PTU modulated retinoic acid regulation of extraocular muscle development and neural crest development. We were able to abrogate the need for PTU by crossing the transgenic lines into a *roy* background, thereby creating the *roy* Tg(*sox10::EGFP*) and *roy* Tg(*α-actin::EGFP*) strains. In the absence of PTU by using the *roy* Tg(*α-actin::EGFP*) strain, treatment with 10 µM DEAB at 24 hpf inhibited pharyngeal arch formation but had minimal effect on jaw and extraocular muscle development at 72 hpf (Figure 1A,B compared to Figure 1I,J) [20]. In the presence of 0.003% PTU which was added to the media at 12 hpf, treatment with 10 µM DEAB at 24 hpf caused thickening and overlapping of extraocular muscles as well as poor formation and occasional absence of the oblique muscles (Figure 1C,D) Importantly addition of only 0.003% PTU at 12 or 22 hpf did not alter craniofacial development (Figure 1K,L compared to Figure 1I,J; Figure S1D compared to Figure S1C; Figure S2C,D compared to Figure 1I,J) at 72 hpf. Addition of 0.003% PTU after 22 hpf did not alter the DEAB-induced extraocular muscle phenotype (Figure S2A,B and S2C,D, compared to Figure 1A,B). This DEAB-induced extraocular muscle phenotype in the presence of PTU could not be fully recapitulated in the *roy* background in the absence of PTU by increasing the concentration of DEAB (15–20 µM; Figure S2E,F) or initiating treatment with DEAB at 18 hpf (Figure S2G,H). Higher concentrations of DEAB started at 24 hpf in combination with PTU at 12 hpf resulted in death by 96 hpf.

We next tested whether the effect of exogenous retinoic acid on craniofacial development was also modulated by PTU. In the *roy* background, treatment with exogenous retinoic acid (100 nM) starting at 28 hpf suppressed neural crest-derived jaw and pharyngeal arch formation by 72 hpf (Figure S1A compared to Figure S1C) and craniofacial muscle development (Figure 1E,F compared to Figure 1I,J). Addition of 0.003% PTU to the media at 12 hpf effectively decreased the teratogenic effects of exogenous retinoic acid (100 nM) as pharyngeal arch formation was incompletely inhibited (Figure S1B compared to Figure S1A). In addition, primitive jaw muscles that were not connected in the midline (Figure 1G,H) were observed in embryos treated with retinoic acid in the presence of 0.003% PTU.

**Figure 2:**
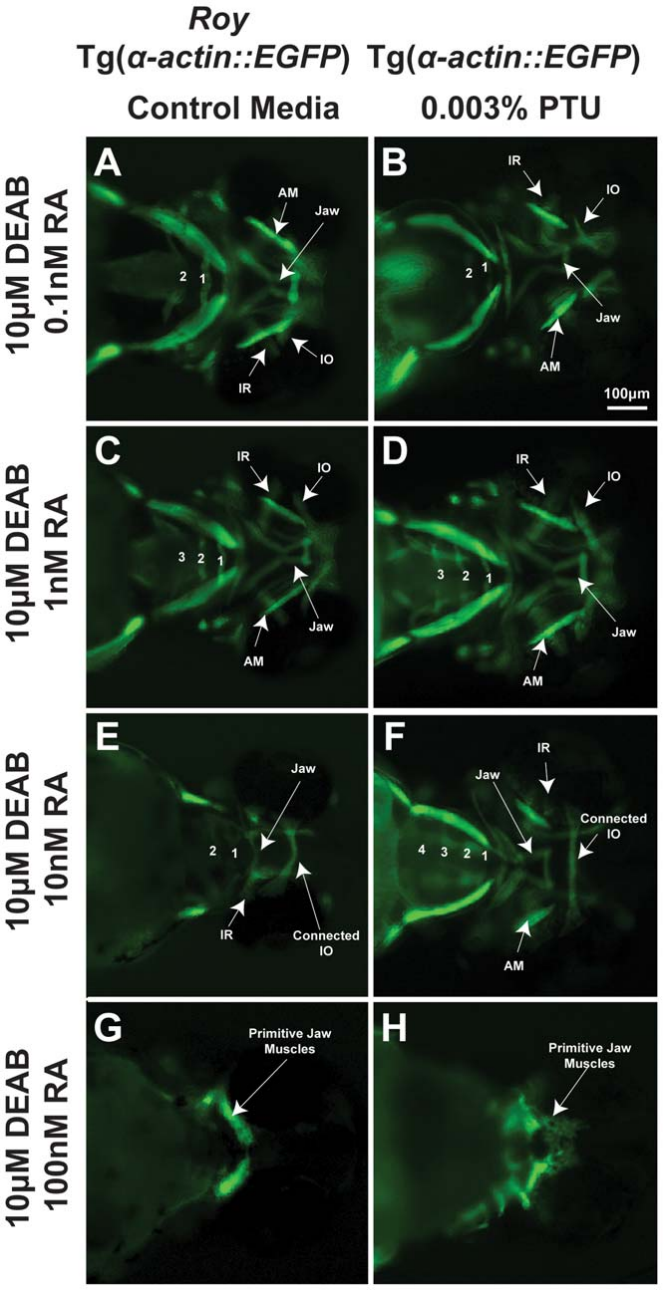
PTU alters sensitivity of craniofacial tissues to retinoic acid. 72 hpf Tg(*α-actin::EGFP*) embryos (ventral view) were treated with 10 µM DEAB at 24 hpf and increasing concentrations of retinoic acid (RA; 0.1 nM, 1 nM, 10 nM, 100 nM) at 28 hpf in the *roy* background (in the absence of PTU; A, C, E, G) or presence of 0.003% PTU added at 12 hpf (B, D, F, H). In the *roy* background and in the absence of PTU, 1 nM retinoic acid improved the DEAB-induced effects on pharyngeal arch formation (C), but at higher concentrations (10 nM (E) and 100 nM (G)), retinoic acid suppressed pharyngeal arch development. DEAB-induced extraocular muscle disorganization in the presence of PTU (Figure 1A, B) was rescued by 0.1 nM retinoic acid (B). The DEAB effects on pharyngeal arch development in the presence of PTU was improved by 1 nM (D) and rescued by 10 nM retinoic acid (F). Teratogenic effects of retinoic acid on jaw musculature and pharyngeal arches were lessened in the presence of PTU (F, H compared to E, G). IR, inferior rectus; IO, inferior oblique; AM, anterior mandibulae.

Since PTU altered the effects of both inhibition of synthesis and treatment with retinoic acid on craniofacial development, we assessed whether PTU modulated the sensitivities to retinoic acid of the jaw and pharyngeal arches. We determined the effect of PTU on the rescue of DEAB-induced defects by exogenous retinoic acid. In the *roy* background, treatment with 0.1 nM retinoic acid at 28 hpf did not rescue the effects 10 µM DEAB at 24 hpf on the pharyngeal arches or jaw (Figure 2A compared to Figure 1A). Retinoic acid at 1 nM improved DEAB-induced defects in pharyngeal arch and jaw formation (Figure 2C compared to Figure 1A). Retinoic acid at 10 nM and 100 nM, not only reversed the effects of 10 µM DEAB but also had teratogenic effects on the jaw and pharyngeal arches (Figure 2E and Figure 2G). The addition of 0.003% PTU at 12 hpf improved pharyngeal arch formation in the presence of a combination of exogenous retinoic acid (0.1 nM, 1 nM, 10 nM, 100 nM) and 10 µM DEAB (Figure 2B,D,F,H compared to Figure 1G and Figure 2A,C,E,G). Furthermore, 0.003% PTU decreased the teratogenic effect of exogenous retinoic acid on jaw development (Figure 2H compared to Figure 2G). Thus, treatment with 0.003% PTU at 12 hpf effectively lowered craniofacial sensitivity to retinoic acid.

### PTU Modulates IGF Regulation of Craniofacial Development

Once we determined that PTU modulates retinoic acid regulation of craniofacial and extraocular muscle development, we also investigated whether other signaling pathways may be affected by PTU. Insulin-like growth factor (IGF) is a ubiquitous and evolutionarily conserved pathway that regulates cellular proliferation, differentiation, migration, and survival [21]. IGF predominantly signals through IGF1 receptor (IGF1R), which in zebrafish has undergone gene duplication (*igf1ra* and *igf1rb*). Previous studies have demonstrated that morpholino oligonucleotide knockdown of each receptor individually caused mild growth and developmental delay by 24 hpf. Knockdown of both *igf1ra* and *igf1rb* resulted in severe growth and developmental inhibition [14].

**Figure 3:**
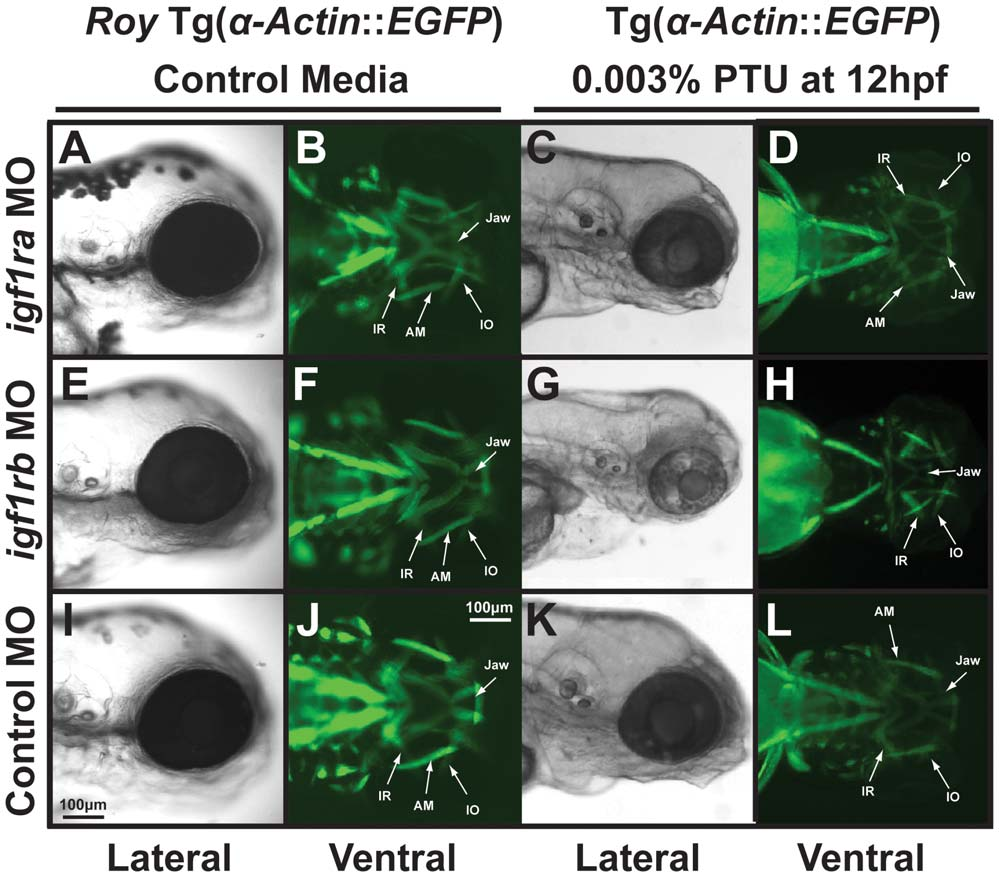
PTU alters IGF regulation of craniofacial muscle development. 72 hpf Tg(*α-actin::EGFP*) embryos that were injected with morpholinos against *igf1ra* (A–D), *igf1rb* (E–H), or control (*globin*; I–L) and were raised in control media (A, B, E, F, I, J) or with 0.003% PTU at 12 hpf (C, D, G, H, K, L). Morpholino knockdown of *igf1ra* or *igf1rb* in the *roy* background (in the absence of PTU; A, B, E, F) did not disrupt craniofacial development as compared to control (I, J). Morpholino knockdown of *igf1ra* in the presence of 0.003% PTU caused developmental delay only in the presence of 0.003% PTU (C, D compared to K, L). Knockdown of *igf1rb* inhibited jaw formation and caused mild thickening of extraocular muscles in the presence of 0.003% PTU (G, H), but not in control media (E, F). IR, inferior rectus; IO, inferior oblique; AM, anterior mandibulae.

IGF1R signaling is also thought to participate in the pathogenesis of thyroid eye disease, in which neural crest-derived orbital fibroblasts undergo proliferation and transdifferentiation [22]. Given our interest in craniofacial and periocular disorders involving neural crest-derived tissues, we studied the effect of IGF signaling on craniofacial and extraocular muscle development. In the *roy* background and in the absence of PTU, morpholino knockdown of *igf1ra* (1 nl of 0.25 mM) or *igf1rb* (1 nl of 0.25 mM), either alone or together caused minimal difference in craniofacial structure (Figure 3A,E compared to Figure 3I) including cartilage (Figure S3A,C,E compared to Figure S3G) and muscle formation at 96 hpf (Figure 3B,F compared to Figure 3J). In the presence of 0.003% PTU added at 12 hpf, knockdown of *igf1ra* caused delay (Figure 3C compared to Figure 3K) of normal neural crest-derived jaw and pharyngeal arch development (Figure S3B compared to Figure S3H) and jaw and extraocular muscle formation (Figure 3D compared to Figure 3L), but the overall morphology was normal. However, addition of 0.003% PTU at 12 hpf to morpholino knockdown of *igf1rb* disrupted jaw formation (Figure S3D, Figure 3G,H) and caused slightly thickened extraocular muscles (Figure 3H), revealing subfunctionalization of igf1r paralogs. Injection of a combination of both *igf1ra* (0.1 mM) and *igf1rb* (0.1 mM) morpholinos in the presence of 0.003% PTU starting at 12 hpf resulted in severely malformed embryos that had gross heart and yolk sac edema, maldeveloped jaw and pharyngeal arches, and shortened body and tail at 96 hpf (Figure S3F, data not shown). Using concentrations of *igf1ra* and *igf1rb* morpholino that were used in the single knockdown experiments (0.25 mM each) resulted in death by 30 hpf as previously described (data not shown) [14].

**Figure 4:**
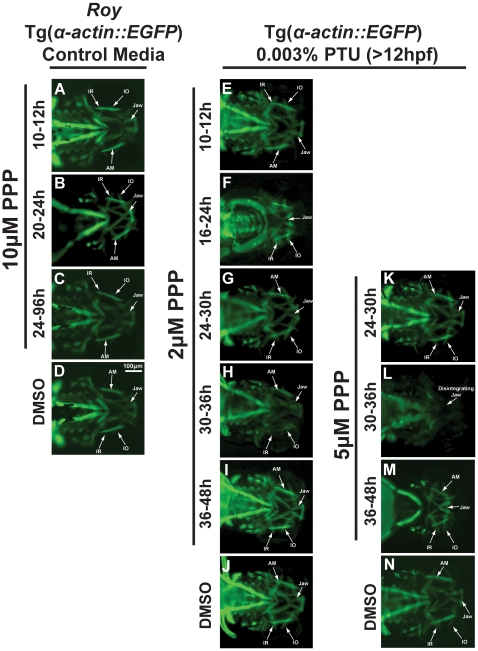
Craniofacial development is sensitive to IGF signaling and PTU during different time frames. 72 hpf Tg(*α-actin::EGFP*) embryos were treated with the tyrosine kinase receptor inhibitor, picropodophyllin (PPP; 2–10 µM) at different times in the *roy* background (in the absence of PTU; A–D) or in the presence of 0.003% PTU added at 12 hpf (E–K). Treatment with PPP between 10–12 hours had minimal effect on craniofacial development (A, E) regardless of treatment with 0.003% PTU. In the *roy* background and in the absence of PTU, treatment with 10 µM PPP between 20 and 24 hpf (B), or 24 and 96 hpf (C) did not affect craniofacial development compared to 0.1% DMSO control (D). Treatment with 2 µM PPP between 16 and 24 hpf in the presence of 0.003% PTU caused craniofacial maldevelopment and thickening of extraocular muscles (F) compared to 0.1% DMSO control (J). Treatment with 2 µM or 5 µM PPP between 24 and 30 hpf caused mild delay of jaw development in the presence of PTU (G). Treatment with 2 µM PPP between 30 and 36 (H) or 36 and 48 (I) hpf did not affect craniofacial development. Treatment with 5 µM PPP between 30 and 36 hpf in the presence of PTU caused poor adherence of the jaw (G) and dissolution of muscle structure after tissue manipulation (L) compared to 0.1% DMSO control (N). Treatment with 5 µM PPP from 36 to 48 hpf caused abnormal jaw and pharyngeal arch formation in the presence of 0.003% PTU (M). Treatment with PPP in the absence of PTU did not affect craniofacial development (M–T). IR, inferior rectus; IO, inferior oblique; AM, anterior mandibulae.

In order to identify specific stages during which IGF signaling is required for craniofacial development, we used picropodophyllin (PPP), an inhibitor of IGF1R tyrosine kinase receptor phosphorylation, which was added to the embryo media at different time-points. Similar to the morpholino knockdown experiments, we found that the PPP-induced phenotype was altered by the addition of 0.003% PTU at 12 hpf. In the *roy* background and in the absence of PTU, treatment with 10 µM PPP at different time points (10–12 hpf, 20–24 hpf, or 24–96 hpf) did not disrupt embryonic development (Figure 4A–C compared to Figure 4D,). Higher concentrations of PPP (up to 100 µM) likewise did not affect craniofacial development (data not shown). The only finding in the absence of PTU was that PPP treatment from 10–12 hpf caused decreased skin pigmentation (data not shown). In the presence of 0.003% PTU added at 12 hpf, embryonic development was sensitive to PPP at concentrations of 2–5 µM, and higher concentrations resulted in death (data not shown). In the presence of 0.003% PTU, treatment with 2 µM PPP from 10–12 hpf did not alter craniofacial development (Figure 4E compared to Figure 4J). However, treatment with 2 µM PPP from 16 to 24 hpf caused jaw and pharyngeal arch malformation with thickening of extraocular muscles at 96 hpf (Figure 4F compared to Figure 4J). A concentration of 5 µM PPP applied at either 10 to 12 hpf or 16 to 24 hpf caused death when added in combination with 0.003% PTU. Treatment with 2 µM PPP from 24 to 30 hpf, 30 to 36 hpf, or 36 to 48 hpf caused minimal disruption of craniofacial development (Figure 4G–I) in the presence of 0.003% PTU. Treatment with 5 µM PPP from 24 to 30 hpf in the presence of 0.003% PTU did not affect craniofacial development (Figure 4G compared to Figure 4N). Treatment with 5 µM PPP from 30 to 36 hpf in the presence of 0.003% PTU resulted in maldeveloped jaws in which craniofacial cartilages failed to adhere to each other (Figure 4L compared to Figure 4N); minor manipulation of these embryos dislodged the jaw, and the craniofacial musculature was disorganized and also poorly connected. Treatment with 5 µM PPP from 36 to 48 hpf in the presence of 0.003% PTU caused a malformed jaw that did not protrude at 96 hpf (Figure 4M compared to Figure 4N). Taken together, these results demonstrate that 0.003% PTU renders craniofacial development dependent on IGF signaling, possibly by inhibiting a redundant pathway.

### Effects of PTU on craniofacial development are dependent on concentration and developmental stage

**Figure 5:**
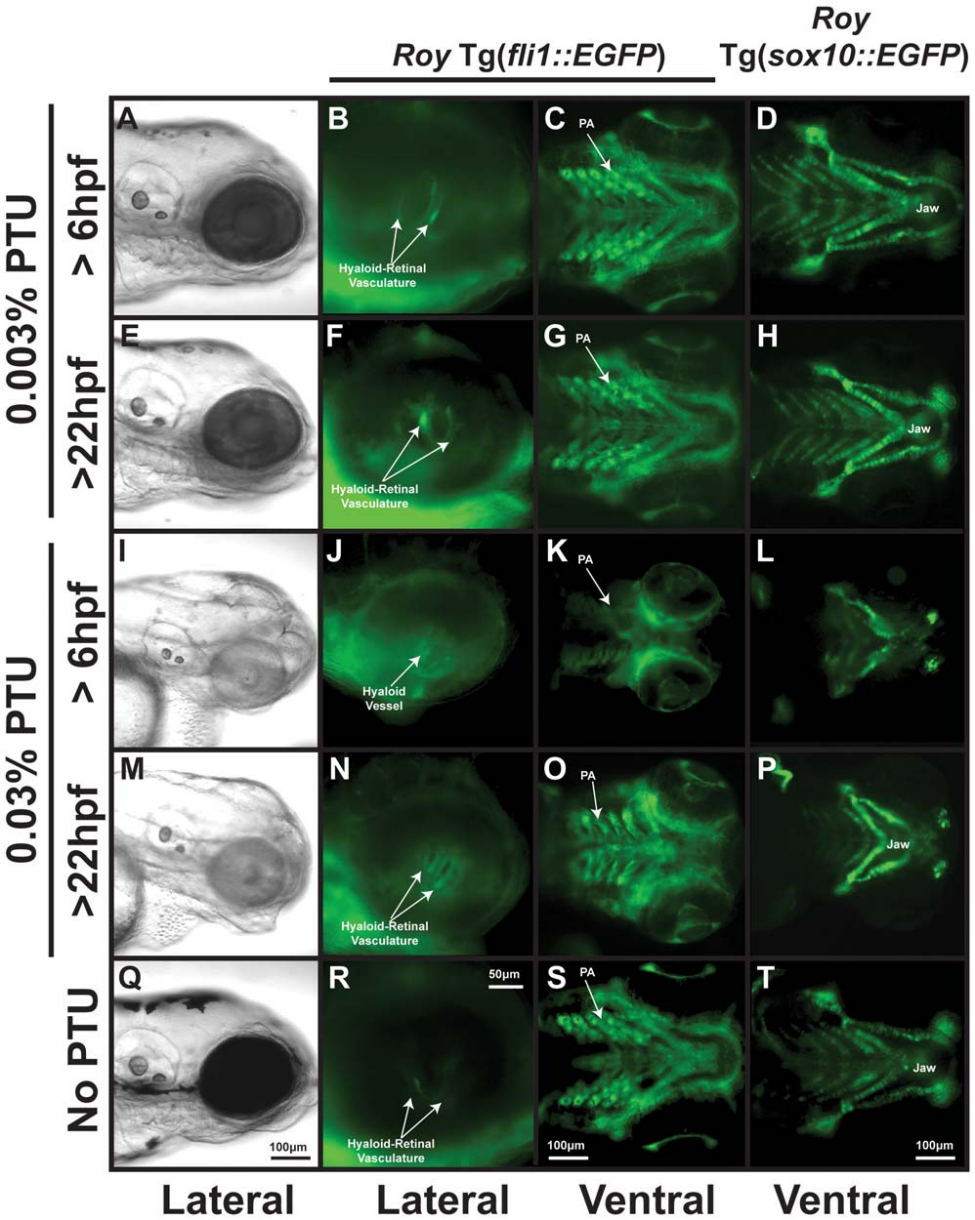
PTU at high concentrations inhibits neural crest development. 96 hpf *roy* Tg(*fli1::EGFP*) and *roy* Tg(*sox10::EGFP*) embryos were treated with 0.003% (A–H) or 0.03% (I–P) PTU at 6 hpf or 22 hpf. Treatment with 0.003% PTU at 6 (A–D) or 22 (E–H) hpf inhibited pigmentation (A, E) but did not affect development of the retinal-hyaloid vasculature (B, F), pharyngeal arches (PA; C, G), and jaw cartilage (D, H) as compared to untreated embryos (Q–T). At a concentration of 0.03%, PTU at 6 hpf inhibited neural crest development (I–L) including pharyngeal arch development (K), jaw formation (L), and the remodeling of the hyaloid vessel into the hyaloids retinal plexus (J). Treatment with 0.03% PTU at 22 hpf caused pharyngeal arch (O) and jaw malformation (M, P), but did not affect the hyaloid-retinal vasculature (N).

PTU at a concentration of 0.003% was previously shown to cause mild teratogenesis and delayed hatching [3], with a dose-dependent response leading to severe malformations and death at higher concentrations [9]. Given the interaction between PTU, retinoic acid and IGF1R in the regulation of craniofacial development, we decided to test whether PTU itself had toxic effects on the neural crest in two well-established transgenic strains, Tg(*fli1::EGFP*) or Tg(*sox10::EGFP*). We compared the toxic effects of PTU (0.003% and 0.03%) on embryos treated at 6 or 22 hpf. Treatment with 0.003% PTU at 6 or 22 hpf caused delayed hatching (data not shown) but overall did not affect craniofacial development (Figure 5A–H) compared to 96 hpf untreated Tg(*fli1::EGFP*) or Tg(*sox10::EGFP*) crossed into the *roy* background (Figure 5Q–T). This included the formation of hyaloid-retinal blood vessels (Figure 5B,F compared to Figure 5R) in the eye, or neural crest-derived pharyngeal arches (Figure 5C,G compared to Figure 5S) or jaw cartilages (Figure 5D,H compared to Figure 5T). Embryos treated at 22 hpf with 0.003% PTU showed more pigment at 96 hpf (Figure 5E) than those treated at 6 hpf (Figure 5A). Treatment with a 10-fold increase in PTU (0.03%) at 6 hpf inhibited growth and development (Figure 5I) and resulted in near-total suppression of neural crest development (Figure 5I–L). In addition, the hyaloid vessels developed but did not remodel to form the retinal-hyaloid vasculature (Figure 5J). Treatment with 0.03% PTU at 22 hpf resulted in malformed jaw (Figure 5M,P) and pharyngeal arches at 96 hpf (Figure 5O,P), but did not affect hyaloid-retinal vasculature formation (Figure 5N). Thus, the toxic effects of PTU are dependent on the concentration and developmental stage at which it is added to the media.

### Effects of PTU on craniofacial development are partially mediated by disruption of thyroid signaling

Previous studies demonstrated that treatment with 0.003% PTU starting at 6 hpf decreased thyroxine (T4) in thyroid follicles of the nasopharynx of 5 days post fertilization (dpf) embryos [9]. The role of thyroid hormone on neural crest development is not well defined, despite the expression of thyroid receptor (TR) αA and αB early in the developing neural crest [23]. Prior to 60 hpf, the yolk sac contains maternal stores of thyroid hormone and is the predominant source [9], [24]. The first evidence of thyroid follicles and endogenous triiodothyronine (T3) and T4 production occurs at about 60 hpf. Zebrafish is an established model for studying the effects of environmental and medicinal goitrogens on thyroid signaling and development of thyroxine-producing follicles [9]. Since zebrafish embryos are most sensitive to PTU when it is added prior to 22 hpf, we investigated whether these toxic effects are mediated through inhibition of thyroid hormone signaling.

**Figure 6:**
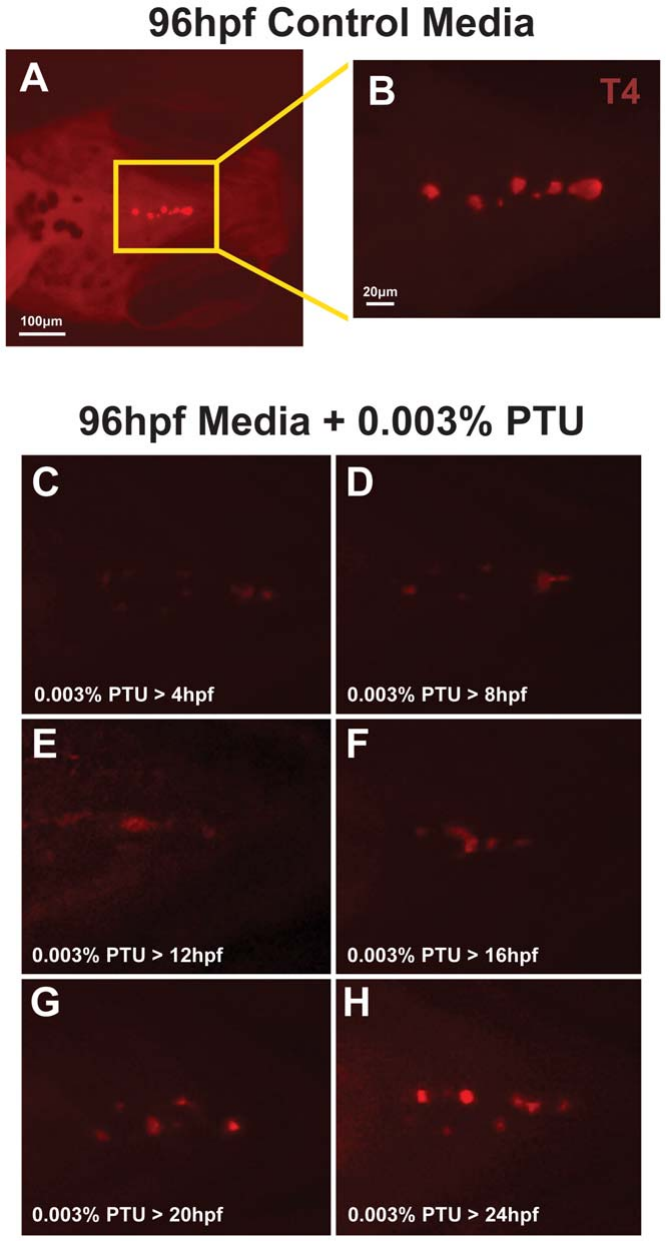
0.003% PTU Inhibits T4 in thyroid follicles. Wholemount immunostaining for T4 in 96 hpf embryos (ventral view) raised in control media (A, B) or in media containing 0.003% PTU. PTU was added at 4, 8, 12, 16, 20, or 24 hpf and carried through until the embryos were harvested at 96 hpf. Untreated 96 hpf embryos showed numerous thyroid follicles that contained T4 (A, B). Addition of 0.003% PTU between 4 and 12 hpf suppressed T4 in follicles at 96 hpf (C, E). Addition of 0.003% PTU between 16 and 20 hpf progressively increases T4 staining and treatment at 24 hpf does not affect T4.

We first used wholemount immunostaining to detect T4 in thyroid follicles scattered in the nasopharynx in embryos raised in control media (Figure 6A,B) and those treated with 0.003% PTU. As previously demonstrated, early treatment (4 to 12 hpf) with 0.003% PTU decreased staining of T4 in thyroid follicles (Figure 6C–E). Treatment with 0.003% PTU between 16 and 24 hpf resulted in more conspicuous T4 staining in the follicles (Figure 6F,G) until no effect was noted when PTU treatment began at 24 hpf (Figure 6H). This suggests that the time frame of PTU-inhibition of thyroid follicle development correlates with the effects of PTU on retinoic acid or IGF signaling.

**Figure 7:**
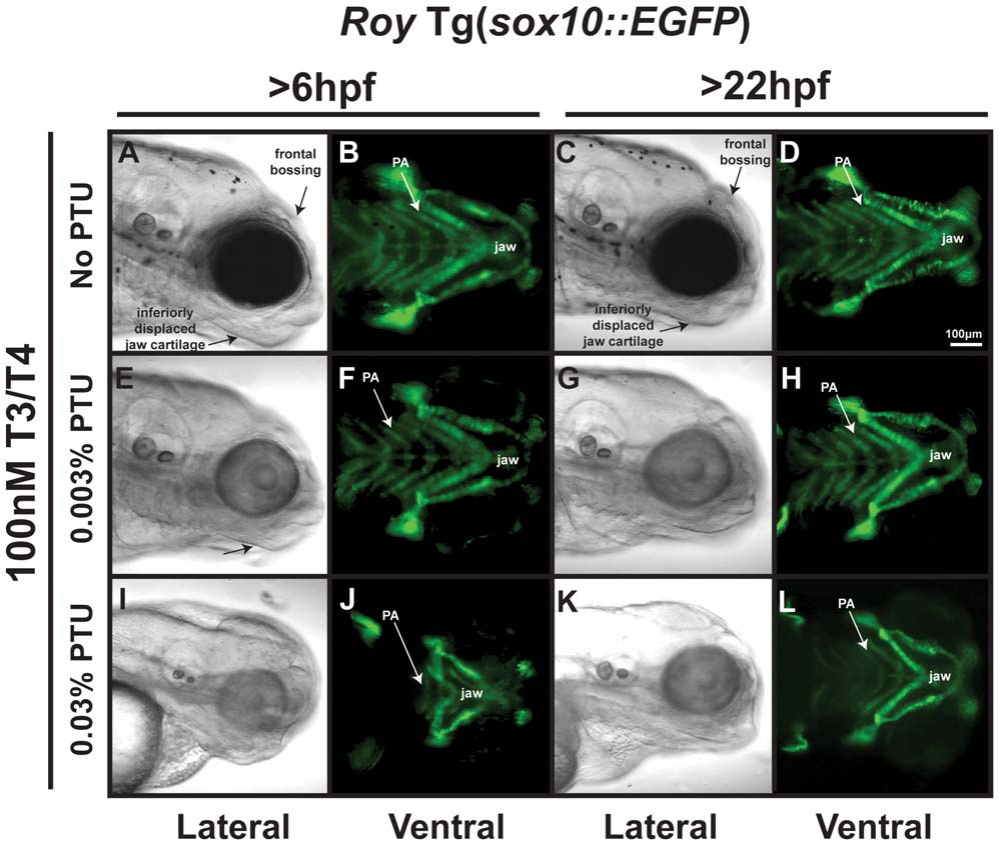
Thyroid hormone partially mediates PTU effect on neural crest. 96 hpf *roy* Tg(*sox10::EGFP*) embryos were treated with 100 nM T3/100 nM T4 and 0.003% (E–H) or 0.03% (I–L) PTU at 6 hpf or 22 hpf. High concentrations of T3 and T4 in the absence of PTU caused thickening of jaw cartilage and frontal bossing (A–D)) when added at 6 or 22 hpf. Treatment with 0.003% PTU lessened the effects of exogenous T3 and T4 (E–H compared to I–L). 100 nM T3/100 nM T4 improved, but did not restore the neural crest defects induced by treatment with 0.03% PTU at 6 (I, J compared to Figure 5I, L) or 22 hpf (K, L compared to Figure 5M, P). PA, pharyngeal arches.

To further investigate whether inhibition of thyroid signaling mediates the effect of PTU on neural crest development, we tested whether PTU defects were rescued with exogenous T3 and T4. *Roy* Tg(*sox10::EGFP*) embryos treated with exogenous 100 nM T3 and 100 nM T4 starting at 6 or 22 hpf showed mild downward displacement of jaw cartilage, frontal bossing, and decreased pigmentation (Figure 7A–D) at 96 hpf. Exogenous T3 and T4 in combination with 0.003% PTU starting at 6 or 22 hpf until 96 hpf resulted in mild displacement of jaw cartilage and frontal bossing (Figure 7E–H compared to Figure 5A,D,E,H). T3 and T4 improved, but did not rescue neural crest-derived jaw development in embryos treated with 0.03% PTU at 6 hpf (Figure 7I,J compared to Figure 5I,L) or 22 hpf (Figure 7K,L compared to Figure 5M,P). Addition of exogenous 100 nM T3 and 100 nM T4 only between 6 and 22 hpf did not affect craniofacial development in the absence of PTU (Figure S4A,B) or in the presence of 0.003% PTU added at 6 hpf (Figure S4C,D). Furthermore, exogenous T3 and T4 between 6 and 22 hpf did not improve neural crest defects induced by treatment with 0.03% PTU from 6 to 96 hpf (Figure S4E,F compared to Figure 5I,L). Taken together, these results suggest that PTU effect on neural crest development may be partly due to inhibition of thyroid hormone signaling.

We next determined whether exogenous T3 and T4 rescued PTU-suppression of T4 in thyroid follicles in 96 hpf embryos. T4 staining was decreased in embryos treated with 0.003% or 0.03% PTU at 6 hpf (Figure S5D,G) compared to untreated embryos (Figure S5A). Treatment with 100 nM T3 and 100 nM T4 between 6 and 22 hpf did not effect T4 staining in 96 hpf embryos that were raised in the absence (Figure S5B) or presence of PTU (0.003% or 0.03%; Figure S5E,H). Treatment with T3 and T4 at 6 hpf did not rescue the effect of 0.003% PTU on T4 staining (Figure S5F), but did mildly improve T4 staining in embryos treated with 0.03% PTU (Figure S5I). Taken together these results demonstrate that PTU disrupts thyroid hormone signaling and endogenous production of thyroxine, and this pathway may be involved in mediating the effects of PTU on neural crest development.

## Discussion

PTU has long been used to inhibit pigmentation in various amphibian and fish models [3], [5], [6]. As a tyrosinase inhibitor, PTU blocks the synthesis of melanin from tyrosine as well as the intermediates required for catecholamine synthesis. In addition, PTU has been shown to inhibit thyroid signaling and follicle development, similar to other known goitrogens [9]. In the current study we demonstrate that PTU has sub-threshold effects on craniofacial development in addition to the notable decreased pigmentation, particularly vis-à-vis retinoic acid and IGF1R signaling.

Retinoic acid gradients in the craniofacial region are critical for ocular, jaw and pharyngeal arch development [15], [16], [17], [20]. We found that the addition of 0.003% PTU at 12 hpf, but not 22 hpf, altered the effects of both retinoic acid deficiency and excess on these structures, as well as on extraocular muscle development. Extraocular muscle development is mediated by signaling and genetic pathways that are distinct from those of branchiomeric muscles (muscles of mastication) or somitic muscles [25], and are generally poorly understood. Studies in zebrafish and chick have shown that signals arising from the developing eye and the cranial neural crest development are required for extraocular muscle morphogenesis [26], [27], [28] and that the homeobox gene *pitx2* appears to be central to these interactions [20]. Retinoic acid, which is produced by the eye at the time of cranial neural crest migration and just prior to extraocular muscle development, is a known regulator of *pitx2* in both mouse [29] and zebrafish [20]. *Pitx2* is required for activating muscle-specific transcription factors such as *Myf5*, *Myog*, *Myod1*, *Smyd1*, *Msc* and *Csrp3* in the prechordal mesoderm [30], [31], and mice deficient for *Pitx2* lack extraocular muscles [32]. Morpholino knockdown of *pitx2a* in zebrafish, unlike mice, did not disrupt extraocular muscle development [20]. This species difference may be due multiple *pitx2* isoforms and the possibility that *pitx3* may functionally substitute for *pitx2a* in the zebrafish. However, the addition of 0.003% PTU caused thickened and disorganized extraocular muscles in the *pitx2* morpholino knockdown (unpublished data), which was similar to the effect of PTU in the context of reduced retinoic acid signaling. Thus, PTU effects on the periocular mesenchyme render extraocular muscle development (and possibly other nearby structures) more susceptible to retinoic acid deficiency.

In addition to its effects on extraocular muscles, we found that PTU modulated neural crest development in conjunction with disruption of IGF signaling. IGF is required for normal embryogenesis [21], and disruptions in IGF signaling have been associated with oncogenesis and disease [33], [34], [35]. In the orbit, IGF1R activation has been implicated in stimulating the pathologic behavior of neural crest-derived tissues in thyroid eye disease [22]. Limited information is known about the environmental and genetic factors that predispose people with autoimmune-related alterations in thyroid levels (*i.e.* Graves disease and Hashimoto's thyroiditis) to develop thyroid eye disease. In the current studies, we found that PTU seemed to pattern the neural crest to make it more dependent on IGF signaling. This could suggest that sub-threshold alterations in neural crest patterning due to genetic or environmental influences during early development may lead to increased vulnerability of orbital tissues to alterations in IGF signaling in the pathogenesis of thyroid eye disease.

PTU has previously been shown to inhibit thyroid hormone signaling in zebrafish [9]. Although endogenous thyroid hormone synthesis does not occur until ∼60 hpf in zebrafish embryos, the yolk sac contains maternal stores of T3 and T4 [24], [36], [37]. The thyroid receptors and deiodinase enzymes that regulate synthesis and degradation of thyroid hormone are expressed well before the appearance of thyroid follicles [23], [37], [38]. At a concentration of 0.003%, PTU added prior to 24 hpf decreased thyroxine in thyroid follicles at 96 hpf. Thus, PTU at an early developmental stage interferes with the development of endogenous thyroid hormone production later in embryogenesis. Treatment with high levels of exogenous T3 and T4 improved but did not rescue jaw formation in embryos treated with high concentrations of PTU. Thus, the effect of PTU on the neural crest is partly mediated by thyroid hormone. The aryl hydrocarbon receptor (AHR) is another pathway that may also mediate the toxic effects of PTU, since activation of AHR is associated with craniofacial malformations that include disruption of jaw development [39], [40], [41], [42], [43]. Further studies are needed to determine additional targets of PTU in neural crest and craniofacial development.

Alternate strategies for maintaining transparency of zebrafish embryos that do not involve the use of PTU have included the use of strains that have decreased pigmentation and auto-fluorescence. In the current studies, we used the *roy orbison* strain which lacks iridophores, but does have skin and eye pigmentation [10] and is particularly useful when using transgenic strains that express fluorescent proteins. On the other hand, the *nacre* strain lacks skin melanin but contains iridophores [10]. The embryos are transparent except for the retinal pigment epithelium and are appropriate for non-fluorescent applications such as color reaction wholemount *in situ* hybridization and immunostaining. The double mutant combining the *roy orbison* and *nacre* mutations constitutes the *casper* strain which lacks skin pigmentation and iridophores [10]. The *casper* strain can be used for all of the above mentioned applications. However, in our experience, the *casper* strain is less robust and more difficult to breed and maintain. Unlike treatment with PTU, all of the above mentioned strains maintain pigmentation of the retinal pigment epithelium. The *sandy* mutant, which carries a mutation in the *tyrosinase* gene [8], lacks both skin and eye pigmentation, but has not been a commonly used strain. A recent study used the *sandy* strain to demonstrate that PTU effect on eye size and retinal development was independent of loss of tyrosinase function (Li and Leung, personal communication), suggesting that the *sandy* strain may be especially useful in studying ocular development.

In the current studies, we demonstrate that the commonly used tyrosinase inhibitor PTU latently altered early developmental processes. These effects manifest when combined with pharmacologic or genetic disruption of other (presumably redundant) pathways, affecting neural crest and mesoderm development, and causing craniofacial abnormalities. As a result, we caution the use of PTU in the study of zebrafish development as it may give rise to different phenotypes and mask the true role of the studied genes.

## Supporting Information

Figure S1 **PTU lessens teratogenic effect of retinoic acid on neural crest.** 72 hpf Tg(*sox10::EGFP*) embryos (ventral view) were treated with 100 nM retinoic acid (RA; A,B) at 28 hpf or 0.1% dimethylsulfoxide (DMSO; I–L) in the *roy* background (in the absence of PTU, A, C) or presence of 0.003% PTU (B, D) which was added to the media at 12 hpf. Exogenous retinoic acid inhibited neural crest-derived pharyngeal arch (PA) formation, which was worse in the absence of PTU (B, D compared to A, C).
(TIF)

Figure S2 **Effect of PTU is time sensitive.** 72 hpf Tg(*α-actin::EGFP*) embryos treated with PTU at 22 hpf and 10 µM DEAB (A, B) at 24 hpf showed shortening of the jaw and only 2 pharyngeal arches, but minimal effect on extraocular muscle development compared to 0.1% DMSO (C, D). In the *roy* background and in the absence of PTU, treatment with 15 µM DEAB at 24 hpf (E, F) or 10 µM DEAB at 18 hpf (G, H) also disrupted jaw and pharyngeal arch formation, but did not cause thickening or loss of extraocular muscles.
(TIF)

Figure S3 **PTU alters IGF regulation of neural crest development.** 72 hpf Tg(*sox10::EGFP*) embryos (ventral view) that were injected with morpholinos against *igf1ra* (A, B), *igf1rb* (C, D), *igf1ra/igf1rb* (E, F) or with a control (*globin*; G, H) and were raised in control media (*roy* background, A C, E, G) or media supplemented with 0.003% PTU at 12 hpf (B, D, F, H). Morpholino knockdown of *igf1ra* caused mild developmental delay only in the presence of 0.003% PTU (B compared to A, G, H). Knockdown of *igf1rb* or both *igf1ra* and *igf1rb* inhibited jaw and pharyngeal arch (PA) formation in the presence of 0.003% PTU (D, F), but not in control media (C, E).
(TIF)

Figure S4 **T3 and T4 are required after 22 hpf for rescue of PTU.** 96 hpf *roy* Tg(*α-actin::EGFP*) were treated with 100 nM T3 and 100 nM T4 from 6 to 22 hpf in the absence (A, B) or presence of 0.003% (C, D) or 0.03% (E, F) PTU from 6 to 96 hpf. Exposure to exogenous T3 and T4 only between 6 and 22 hpf in the absence of PTU (A, B) or in the presence of 0.003% PTU (C, D) did not cause frontal bossing or displacement of jaw cartilage as was seen with exposure to T3 and T4 from 6 to 96 hpf (Figure 7A–D). Furthermore, exogenous T3 and T4 from 6 to 22 hpf did not rescue the craniofacial defects induced by 0.03% PTU (E, F).
(TIF)

Figure S5 **T3 and T4 partially restore T4 expression in PTU-treated embryos.** Wholemount immunostaining for T4 in 96 hpf embryos (ventral view) demonstrated that in the absence of PTU exogenous treatment with 100 nM T3 and 100 nM T4 between 6 and 96 hpf mildly decreased T4 expression in thyroid follicles (C) compared to embryos treated with T3 and T4 from 6 to 22 hpf (B) and untreated controls (A). Treatment with 0.003% (D) and 0.03% PTU (G) progressively decreased T4 expression compared to embryos raised in the absence of PTU (A). Treatment with exogenous T3 and T4 from 6 to 22 hpf (E) or from 6 to 96 hpf (F) did not improve the effect of 0.003% PTU on T4 expression (D) while exogenous T3 and T4 from 6 to 96 hpf (I), but not from 6 to 22 hpf (H) slightly improved T4 expression in embryos treated with 0.03% PTU (G).
(TIF)
