# Supplementary figures and images for: Phenothiourea Sensitizes Zebrafish Cranial Neural Crest and Extraocular Muscle Development to Changes in Retinoic Acid and IGF Signaling
Source: PLoS One. 2011 Aug 19;6(8):e22991. doi: 10.1371/journal.pone.0022991 (PMC3158757; doi:10.1371/journal.pone.0022991)

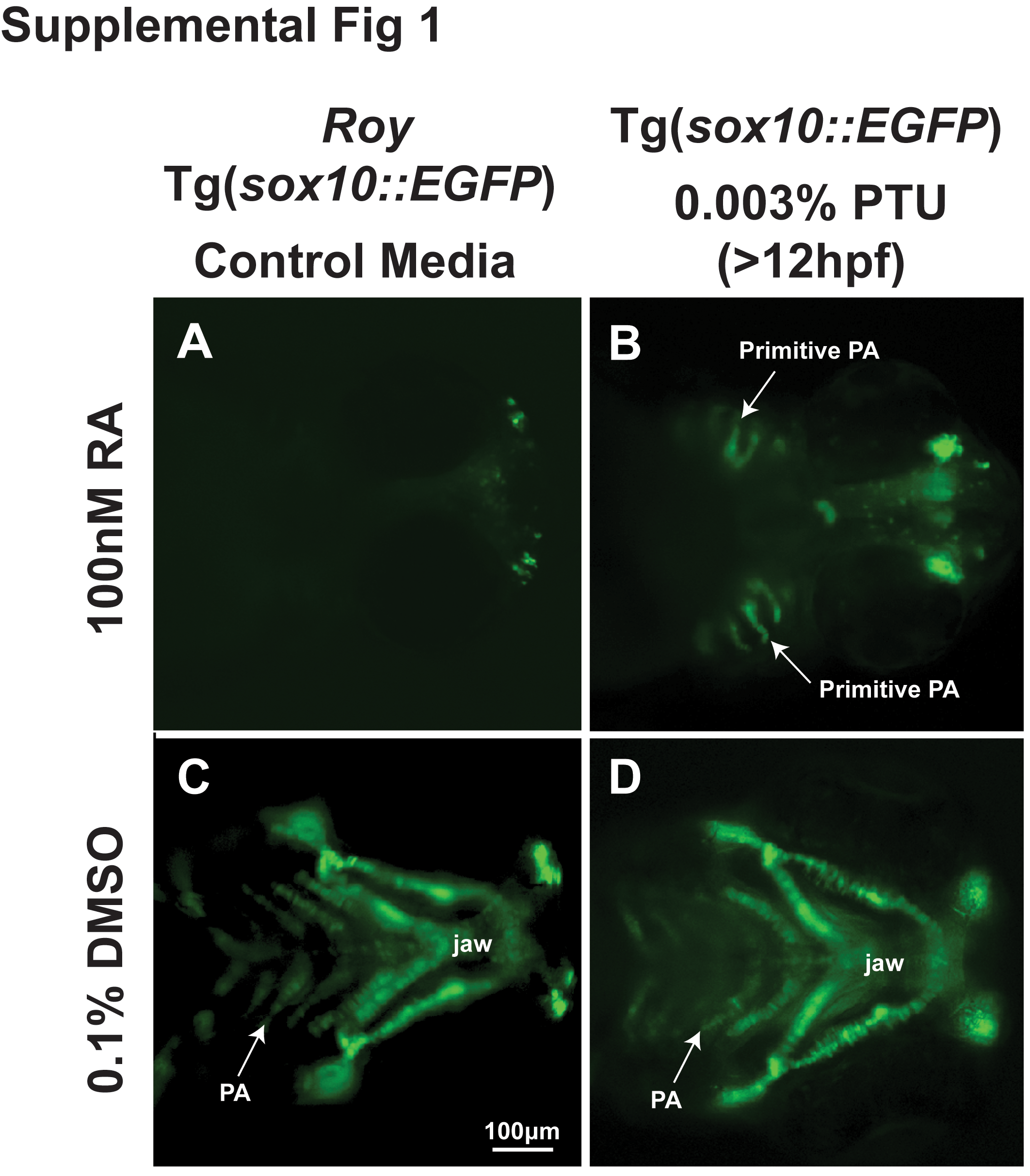

Supplement: Figure S1 — PTU lessens teratogenic effect of retinoic acid on neural crest. 72 hpf Tg(sox10::EGFP) embryos (ventral view) were treated with 100 nM retinoic acid (RA; A,B) at 28 hpf or 0.1% dimethylsulfoxide (DMSO; I–L) in the roy background (in the absence of PTU, A, C) or presence of 0.003% PTU (B, D) which was added to the media at 12 hpf. Exogenous retinoic acid inhibited neural crest-derived pharyngeal arch (PA) formation, which was worse in the absence of PTU (B, D compared to A, C). (TIF) [file pone.0022991.s001.tif]

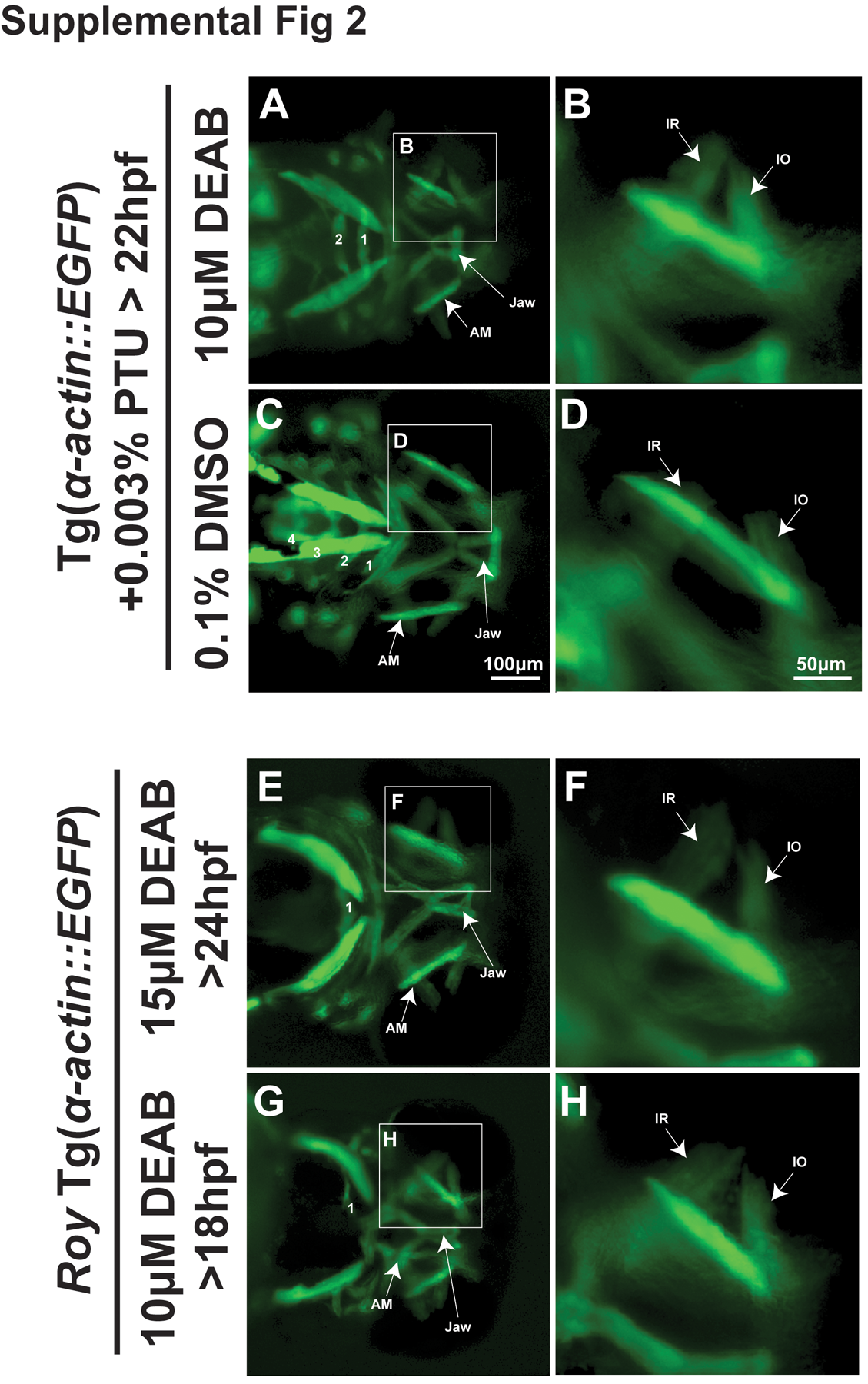

Supplement: Figure S2 — Effect of PTU is time sensitive. 72 hpf Tg(α-actin::EGFP) embryos treated with PTU at 22 hpf and 10 µM DEAB (A, B) at 24 hpf showed shortening of the jaw and only 2 pharyngeal arches, but minimal effect on extraocular muscle development compared to 0.1% DMSO (C, D). In the roy background and in the absence of PTU, treatment with 15 µM DEAB at 24 hpf (E, F) or 10 µM DEAB at 18 hpf (G, H) also disrupted jaw and pharyngeal arch formation, but did not cause thickening or loss of extraocular muscles. (TIF) [file pone.0022991.s002.tif]

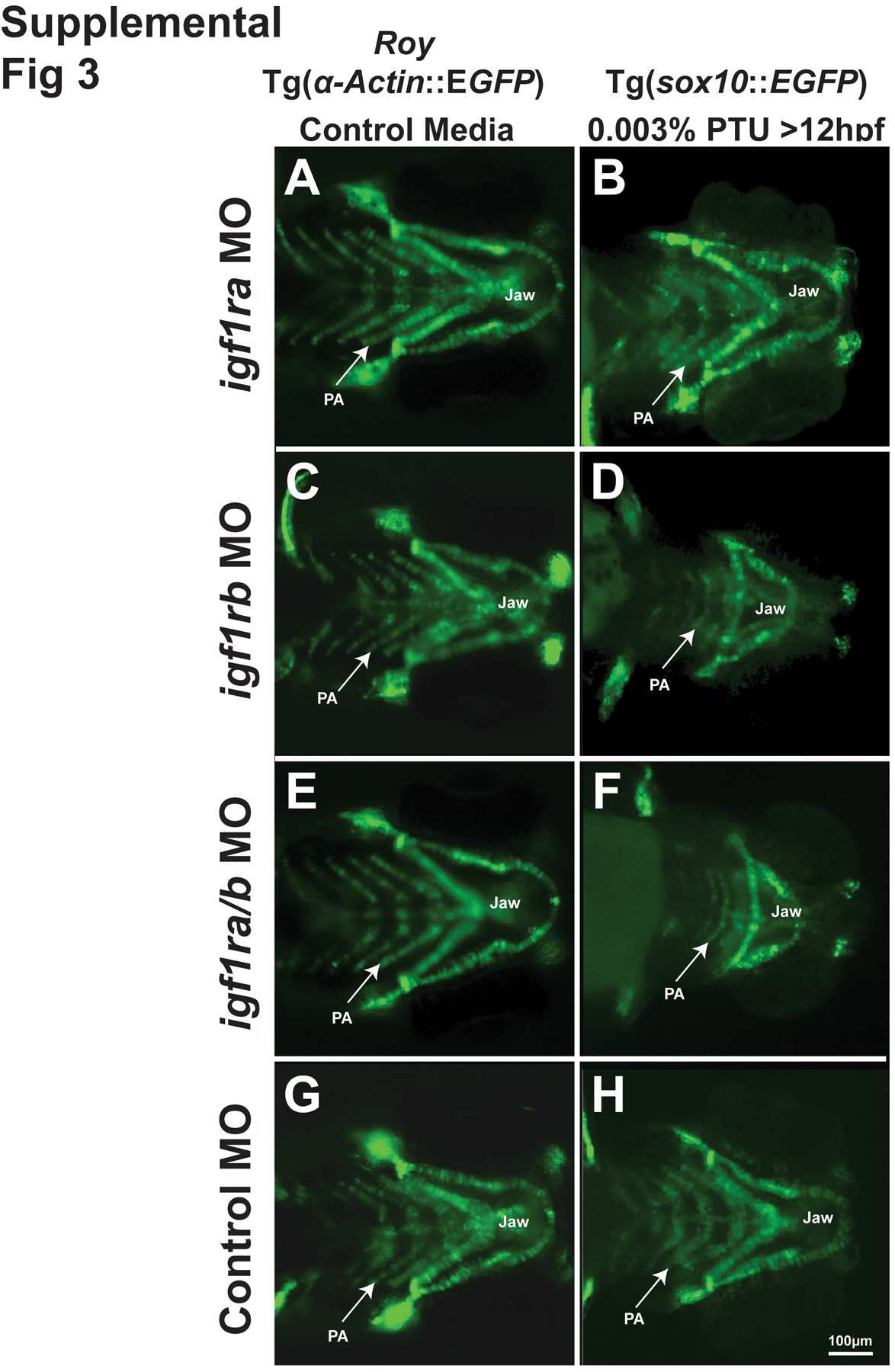

Supplement: Figure S3 — PTU alters IGF regulation of neural crest development. 72 hpf Tg(sox10::EGFP) embryos (ventral view) that were injected with morpholinos against igf1ra (A, B), igf1rb (C, D), igf1ra/igf1rb (E, F) or with a control (globin; G, H) and were raised in control media (roy background, A C, E, G) or media supplemented with 0.003% PTU at 12 hpf (B, D, F, H). Morpholino knockdown of igf1ra caused mild developmental delay only in the presence of 0.003% PTU (B compared to A, G, H). Knockdown of igf1rb or both igf1ra and igf1rb inhibited jaw and pharyngeal arch (PA) formation in the presence of 0.003% PTU (D, F), but not in control media (C, E). (TIF) [file pone.0022991.s003.tif]

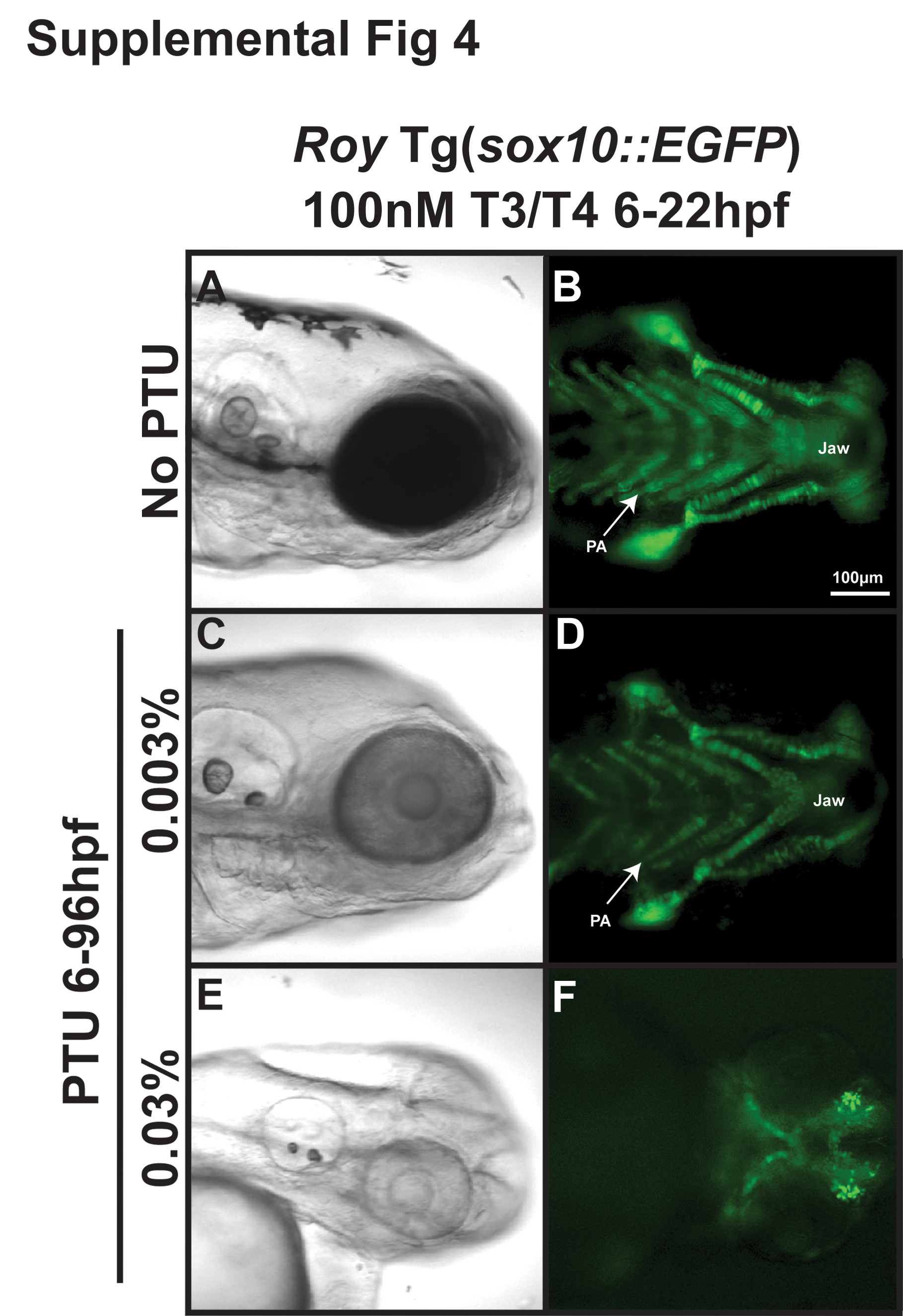

Supplement: Figure S4 — T3 and T4 are required after 22 hpf for rescue of PTU. 96 hpf roy Tg(α-actin::EGFP) were treated with 100 nM T3 and 100 nM T4 from 6 to 22 hpf in the absence (A, B) or presence of 0.003% (C, D) or 0.03% (E, F) PTU from 6 to 96 hpf. Exposure to exogenous T3 and T4 only between 6 and 22 hpf in the absence of PTU (A, B) or in the presence of 0.003% PTU (C, D) did not cause frontal bossing or displacement of jaw cartilage as was seen with exposure to T3 and T4 from 6 to 96 hpf (Figure 7A–D). Furthermore, exogenous T3 and T4 from 6 to 22 hpf did not rescue the craniofacial defects induced by 0.03% PTU (E, F). (TIF) [file pone.0022991.s004.tif]

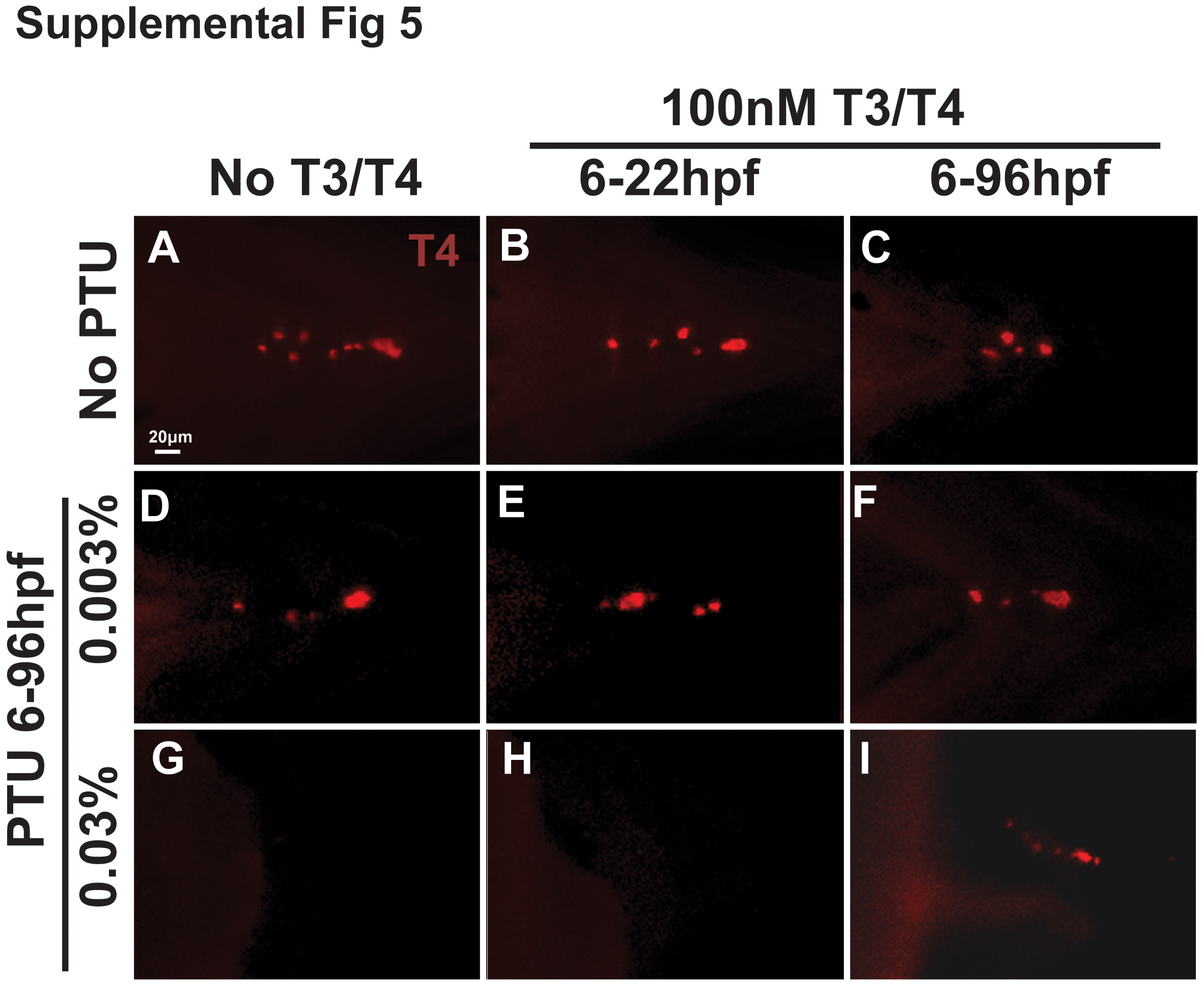

Supplement: Figure S5 — T3 and T4 partially restore T4 expression in PTU-treated embryos. Wholemount immunostaining for T4 in 96 hpf embryos (ventral view) demonstrated that in the absence of PTU exogenous treatment with 100 nM T3 and 100 nM T4 between 6 and 96 hpf mildly decreased T4 expression in thyroid follicles (C) compared to embryos treated with T3 and T4 from 6 to 22 hpf (B) and untreated controls (A). Treatment with 0.003% (D) and 0.03% PTU (G) progressively decreased T4 expression compared to embryos raised in the absence of PTU (A). Treatment with exogenous T3 and T4 from 6 to 22 hpf (E) or from 6 to 96 hpf (F) did not improve the effect of 0.003% PTU on T4 expression (D) while exogenous T3 and T4 from 6 to 96 hpf (I), but not from 6 to 22 hpf (H) slightly improved T4 expression in embryos treated with 0.03% PTU (G). (TIF) [file pone.0022991.s005.tif]
